# Adult spinal opioid receptor μ1 expression after incision is altered by early life repetitive tactile and noxious procedures in rats

**DOI:** 10.1002/dneu.22583

**Published:** 2018-02-28

**Authors:** Nynke J. van den Hoogen, Roel RI van Reij, Jacob Patijn, Dick Tibboel, Elbert A. J. Joosten

**Affiliations:** ^1^ Department of Anaesthesiology and Pain Management Maastricht University Medical Centre Maastricht The Netherlands; ^2^ Department of Translational Neuroscience, School of Mental Health and Neuroscience Maastricht University Maastricht The Netherlands; ^3^ Intensive Care and Department of Paediatric Surgery Erasmus MC‐Sophia Rotterdam, Maastricht The Netherlands

**Keywords:** neonate, spinal cord, procedural pain, post‐operative pain, opioid receptor

## Abstract

Clinical and experimental data suggests that noxious stimulation at critical stages of development results in long‐term changes on nociceptive processing in later life. Here, we use an established, well‐documented rat model of repetitive noxious procedures closely mimicking the clinical situation in the NICU. In order to understand molecular changes underlying the long‐term consequences of repetitive stimulation of the developing nociceptive system the present study aims to analyze the presence of the µ‐opioid‐receptor‐1 (OPRM1). Neonatal rats received either four needle pricks per day in the left hind‐paw from postnatal day 0–7 as a model of procedural pain in infancy. Control pups were handled in the same way but were instead tactile stimulated, or were left undisturbed. At the age of 8 weeks, all animals received an ipsilateral hind‐paw incision as a model for post‐operative pain, and mechanical sensitivity was tested at multiple time‐points. Before, and 1 or 5 days post‐incision, spinal cord tissue was collected for immunostaining of opioid receptor OPRM1. Semi‐quantitative immunocytochemical analysis of superficial laminae in lumbar spinal dorsal horn revealed that: (1) early life repetitive tactile or noxious procedures do not alter baseline levels of OPRM1 staining intensity and (2) early life repetitive tactile or noxious procedures lead to a decrease in OPRM1 staining intensity 5 days after incision in adulthood compared to undisturbed controls. We conclude that early life repetitive tactile or noxious procedures affect the intensity of OPRM1‐immunoreactivity in the lumbar superficial spinal cord dorsal horn after adulthood injury, without affecting baseline intensity. © 2018 The Authors. Developmental Neurobiology Published by Wiley Periodicals, Inc. Develop Neurobiol 78: 417–426, 2018

## INTRODUCTION

Exposure to painful procedures during the early postnatal period leads to altered pain perception in adulthood (Schwaller and Fitzgerald, 2014; Ren et al., 2004; Laprairie and Murphy, 2009). It has been demonstrated that this pain perception in adulthood is related to changes in nociceptive processing in spinal cord and brain (Schwaller and Fitzgerald, 2014; Ruda et al., 2000; Fitzgerald et al., 1994; Walker et al., 2015). More specifically, changes in descending inhibitory control of the local spinal nociceptive network have been identified (Schwaller and Fitzgerald, 2014; Laprairie and Murphy, 2009; Walker et al., 2015). These changes may underlie the observed differences in sensitivity and pain perception in adulthood, as it leads to increased hypersensitivity with re‐injury of the same dermatome. In this respect, further research on the consequences of early life pain on the (descending) inhibitory opioid system is of particular interest, as the opioid system is important in control of the local spinal nociceptive network and also because opioids are the drug of choice when treating acute postoperative pain later in life (Argoff, 2014). Endogenous opioids β‐endorphin and enkephalins (met‐ and/or leu‐enkephalin), as well as pain treatment with opioids like morphine or fentanyl are directly dependent on descending control of the µ‐opioid receptor 1 (opioid receptor mu, OPRM1). The µ‐opioid receptor 1 is the most important opioid receptor responsible for descending pain modulation, and is distributed throughout the central nervous system (CNS) (Basbaum et al., 2009; Fitzgerald and Koltzenburg, 1986; Millan, 2002). For analgesia, the µ‐opioid receptor is among the most important targets. OPRM1 is localized on the pre‐ and postsynaptic membrane of primary and secondary afferents in the nociceptive system and is heavily expressed in the neocortex, striatum, hippocampus, periaqueductal gray and the superficial (laminae I and II) spinal cord dorsal horn (Arvidsson et al., 1995; Fields et al., 2006). In response to an ongoing noxious stimulus, opioid expression is upregulated in supra‐ and in spinal regions, binding at OPRM1 and resulting in a reduction of spinal nociception (Fields et al., 2006; Herz et al., 1989). Neonatally, the opioid system undergoes maturational changes (Fitzgerald and Koltzenburg, 1986; Kornblum et al., 1987; Nandi et al., 2004; Thornton et al., 1998). While the spinal anatomical connections are present at birth, the descending inhibitory control needs functional maturation in the first postnatal weeks before adult levels of functionality are reached (Fitzgerald and Koltzenburg, 1986). The expression of OPRM1 undergoes major postnatal development and as a consequence morphine sensitivity undergoes changes (Nandi et al., 2004; Thornton et al., 1998). Morphine seems to elicit dose‐dependent anti‐nociception in rats from P3 on, but it becomes more potent from P9 on (Thornton et al., 1998). Although µ‐opioid receptors are present in the rat CNS at birth and increase in density to adult levels around postnatal day (P)14–21, it remains unclear when efficient coupling to the G protein units takes place during the early postnatal period, seemingly leading to a decreased morphine response before P9 (Kornblum et al., 1987; Thornton et al., 1998). Stimulation of this maturing descending inhibitory system during early postnatal development leads to chronic alterations (summarized in Schwaller and Fitzgerald, 2014). Animal studies have shown early life inflammatory pain/nociception leads to an elevation of opioids, and a downregulation of opioid receptors in the periaqueductal gray (Laprairie and Murphy, 2009; Laprairie et al., 2008), but the effect of early life pain on the spinal gate is not known. The objective of the present study is to determine the long‐term changes of neonatal repetitive procedural pain on the presence of u‐opioid OPRM1 in the lumbar spinal cord dorsal horn. This subsequently may give important insights into the role and development of descending inhibitory opioid systems in control of the local spinal nociceptive system. Identification of changes in immuno‐cytochemical staining of OPRM1 in lumbar spinal dorsal horn superficial laminae after early life experiences is of major relevance, as post‐surgical pain is mostly treated with opioids in adulthood.

The study aims were twofold; we first aimed to study the effect of neonatal repetitive noxious (needle prick) priming on adult baseline levels of OPRM1 staining intensity in lumbar spinal cord in adulthood. The second aim of the study was to test the effect of neonatal repetitive pain priming on OPRM1 staining intensity after re‐injury of the same dermatome. To achieve this, the staining intensity of OPRM1 in lumbar spinal cord tissue from neonatally primed animals (Knaepen et al., 2013; Valverde et al., 2016) was assessed to test our hypothesis that early life pain leads to a decrease in adult lumbar spinal OPRM1 staining intensity. Furthermore, the neonatal (needle prick) priming model has been shown to result in robust longer lasting hypersensitivity following re‐injury of the same dermatome in adulthood (Knaepen et al., 2013; Valverde et al., 2016). From this we hypothesized that lumbar (L4–L5) spinal cord OPRM1 expression in neonatal needle prick primed animals would be decreased after incision in the adult (as the prolonged hypersensitivity suggests a lack of endogenous pain modulation).

Our study shows that neonatal repetitive procedures, whether tactile or painful, do not change baseline OPRM1 staining intensity in the adult lumbar spinal cord superficial dorsal horn, but neonatal repetitive procedures results in a significantly decreased OPRM1 staining intensity after same dermatome skin injury in the adult rat.

## METHODS

### Animals

Fifty‐eight Sprague‐Dawley rats were used, divided over three groups, as specified in Table 1. All rats were born on the 21st day of gestation from time‐pregnant Sprague‐Dawley dams (Charles River, delivered at 13th day of gestation) at Maastricht University animal facility. Litters were culled to a maximum of 10 pups. At postnatal day (P)21, pups were weaned and housed in same‐sex standard cages with 2–3 littermates in a temperature‐ (19°C–24°C) and humidity‐ (55% ± 15%) controlled room with a reversed 12/12 h day‐night cycle. Food and water was available *ad libitum* for the duration of the study.

**Table 1:** Animal numbers per condition and time point

	Baseline	1 day post‐incision	3 days post‐incision	5 days post‐incision
UC behavior	11 males, 10 females	8 males, 6 females	5 males, 2 females	5 males, 2 females
UC staining	3 males, 4 females	3 males, 4 females	–	5 males, 2 females
TC behavior	10 males, 10 females	5 males, 8 females	1 male, 5 females	1 male, 5 females
TC staining	5 males, 2 females	4 males, 3 females	–	1 male, 5 females
NP behavior	9 males, 8 females	5 males, 6 females	3 males, 4 females	3 males, 4 females
NP staining	4 males, 2 females	2 males, 2 females	–	3 males, 4 females

Rat pups were divided over three groups: neonatally undisturbed control (UC), tactile control (TC), or neonatal needle prick (NP, littermates of TC animals).

All animal experiments were performed in accordance with the European Directive for the Protection of Vertebrate Animals Used for Experimental and Other Scientific Purposes (86/609/EEC) and were approved by the Committee for Experiments on Animals, Maastricht, The Netherlands (DEC 2011‐041/DEC 2014‐090).

### Study Design

**Figure 1:**
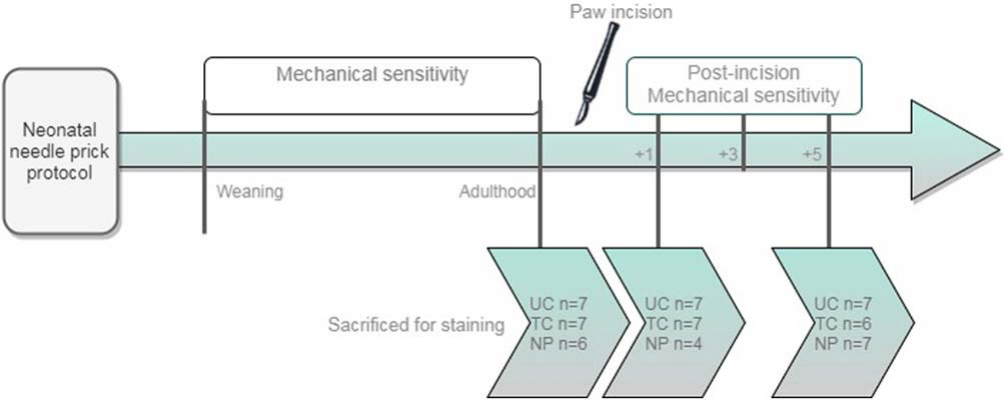
Flowchart of the study design. After undergoing the neonatal needle prick protocol, animals were weaned at postnatal day 21. Mechanical sensitivity was assessed from weaning to adulthood. In adulthood, animals from all three groups were sacrificed for ICC staining purposes (UC *n* = 7, TC *n* = 7, NP *n* = 6). Following paw incision, behavioral assessment of mechanical sensitivity was tested at 1, 3, and 5 days post‐incision. Animals were sacrificed on post‐incision day 1 (UC *n* = 7, TC *n* = 7, NP *n* = 4) and 5 (UC *n* = 7, TC *n* = 6, NP *n* = 7) for ICC staining purposes. Abbreviations: UC: undisturbed control; TC: tactile control; NP: Needle prick; +1: 1 day post‐incision; +3: 3 days post‐incision; +5: 5 days post‐incision. [Color figure can be viewed at http://wileyonlinelibrary.com]

To assess differences in mechanical sensitivity and OPRM1 staining at different time‐points, behavioral testing of mechanical sensitivity was performed before incision, and 1, 3, and 5 days post‐incision. Following behavioral testing, animals were sacrificed at three different days; before undergoing incision, 1 day post‐incision and 5 days post‐incision. The study design is visualized in Figure 1. The experimenters were blinded to neonatal condition during all experimental procedures: the behavioral testing from 3 weeks of age, the immunohistochemistry, microscopy, data processing, and statistical analysis.

### Neonatal Painful Procedures

A neonatal repetitive needle prick model was implemented as described before (Knaepen et al., 2013; van den Hoogen et al., 2016). Briefly, rat pups received four unilateral needle pricks per day from day of birth to P7 in the left hind paw (needle prick group, NP, males *n* = 9, females *n* = 8). Control pups were handled in the same way and received a tactile stimulus by stroking the plantar surface of the paw with a cotton swab (Tactile Control group, TC, males *n* = 10, females *n* = 10), or were left undisturbed (Undisturbed Control group, UC, males *n* = 11, females *n* = 10). This model leads to robust acute hypersensitivity as well as localized mechanical hypersensitivity after re‐injury of the same dermatome in adulthood in the NP group compared to control.

### Adult Mechanical Sensitivity and Ipsilateral Paw Incision

Mechanical sensitivity was assessed during development from weaning to adulthood (P21–56, 3–8 weeks of age) using plantar application of calibrated Von Frey hairs as described previously (van den Hoogen et al., 2016). Briefly, animals were placed in a Plexiglas cage on a mesh floor. Filaments with logarithmically increasing force were applied to the plantar surface of the hind‐paws (1.202; 2.041; 3.63; 5.495; 8.511; 15.136; and 28.84 g) for 5 s using the up–down method described by Chaplan et al. (1994). Mechanical sensitivity was tested each week from 3 to 8 weeks of age. In adulthood (P49–P56) animals received an ipsilateral plantar hind‐paw incision as described earlier (Valverde et al., 2016; Brennan et al., 1996). Briefly, all animals were anaesthetized with isoflurane (4% induction, 2.5% maintenance), followed by a 1‐cm longitudinal incision through the plantar skin and fascia. The underlying plantar muscle was elevated and incised at the midline (∼1–2 mm). Skin was sutured and the animals were placed back in the home cage for recovery. Mechanical sensitivity was assessed as described before at 1, 3, and 5 days post‐incision to assess post‐surgical hypersensitivity (Knaepen et al., 2013).

### Perfusion Fixation, Immunohistochemistry and Image Processing

In adulthood (age 7–8 weeks), all animals were terminally anaesthetized after behavioral testing with sodium pentobarbital (150 mg/kg intraperitoneally) and transcardially perfused with ice‐cold tyrode buffer followed by Somogyi fixative. The lumbar spinal cord was post‐fixed overnight in perfusion solution followed by cryoprotection (10% sucrose solution, overnight, 25% sucrose solution, 72 h). Spinal levels L4 and L5 were frozen and stored at −80°C. 30 µm thick serial Cryostat sections (CM3050 S, Leica, Nussloch, Germany) were mounted on gelatine‐coated glass slides and stored at −20°C. Slides were thawed before washing three times with Tris‐buffered‐saline (TBS), TBS‐T (TBS with Triton‐X (0.2%) and TBS. After overnight incubation at room temperature with primary antibody against OPRM1 (rabbit α‐OPRM1, 1:5000, Immunostar, Inc. Hudson, WI), the slides were washed (3 washes of 10 min, TBS/TBS‐T/TBS) incubated with donkey anti‐rabbit secondary antibody labelled with Alexa 488 (1:100, Invitrogen) for 2 h. Finally, the sections were washed with TBS/TBS‐T/TBS (3 washes of 10 min. each) and embedded in 80% glycerol in phosphate‐buffered‐saline (PBS). Photographs of ipsi‐ and contralateral dorsal horns were taken using a fluorescent microscope (Olympus AX70) with a 10× objective using a grayscale F‐view camera (Olympus). Photographs were analyzed using ImageJ free software. Six sections per lumbar level (L4 or L5) per animal were averaged for each animal; mean gray value of OPRM1 immunoreactivity was determined after manual background subtraction. The superficial dorsal horn (lamina I–III) in lumbar spinal cord L4–L5 was measured using atlas coordinates and delineated as region of interest (ROI) as described before by our group (Knaepen et al., 2013). Gray value was determined using ImageJ software (ImageJ 1.48v, Wayne Rasband National Institutes of Health, USA). To assess whether we stained exclusively neuronal OPRM1, double‐labeling with neuronal marker NeuN was performed on a separate series of slides. For this, staining was performed as above for day 1 and 2, but instead of embedding with glycerol, slides were incubated overnight with a second primary antibody against NeuN (Mouse α‐NeuN, Millipor, 1:50). On day 3, slides were washed with TBS (3× 10 min) and incubated for 2 h with donkey anti‐mouse secondary antibody labelled with Alexa 594 (1:100, Invitrogen), and washed with TBS (3× 10 min). To enable easy recognition of our ROI in slides without photo bleaching in channels 488 and 594, slides were incubated with Hoechst for 30 min. Finally, slides were washed 3× 10 min with TBS and embedded in 80% glycerol in PBS. Photomicrographs were captured using a confocal microscope at 40× magnification (DSU, Olympus).

### Statistical Analysis

Baseline differences in behavior and OPRM1 gray values were analyzed using one‐way ANCOVA with Bonferroni Posthoc testing correction (IBM SPSS Statistics, version 23 for Windows, IBM corp., Armonk, NY). Between and within group differences over time were tested using two‐way ANCOVA with Bonferroni correction (IBM SPSS Statistics, version 23 for Windows, IBM corp., Armonk, NY). For the behavioral analysis, the Von Frey data was log‐transformed to allow for parametric testing. ANCOVA analysis was used to correct for sex, as there are differences in distribution of sexes in different groups. All data is represented as mean ± SEM. A *P* value <0.05 was considered statistically significant.

## RESULTS

### Neonatal Repetitive Tactile and Needle Prick Priming Does Not Alter Baseline Mechanical Sensitivity or Baseline OPRM1 Staining Intensity in the Superficial SCDH

**Figure 2:**
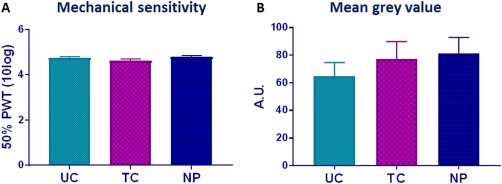
Baseline values of (A) mechanical sensitivity expressed as 50% paw withdrawal threshold (50%PWT) and (B) Superficial (laminae I–III) SCDH OPRM1 staining intensity expressed as mean gray value. Rat pups (littermates) received four noxious needle pricks (needle prick, NP, *n* = 17) or tactile stimulation (tactile control, TC, *n* = 20), or were left undisturbed (undisturbed control, UC, *n* = 21). (A) No differences between group were found (effect of procedure: *F*(2, 57) = 1.615, *P* = 0.208). (B) In adulthood, spinal cord level L4 and L5 was stained for OPRM1. Superficial SCDH intensity of OPRM1 staining was comparable between neonatal procedure groups (one‐way ANOVA). Data is presented as mean ± SEM. [Color figure can be viewed at http://wileyonlinelibrary.com]

**Figure 3:**
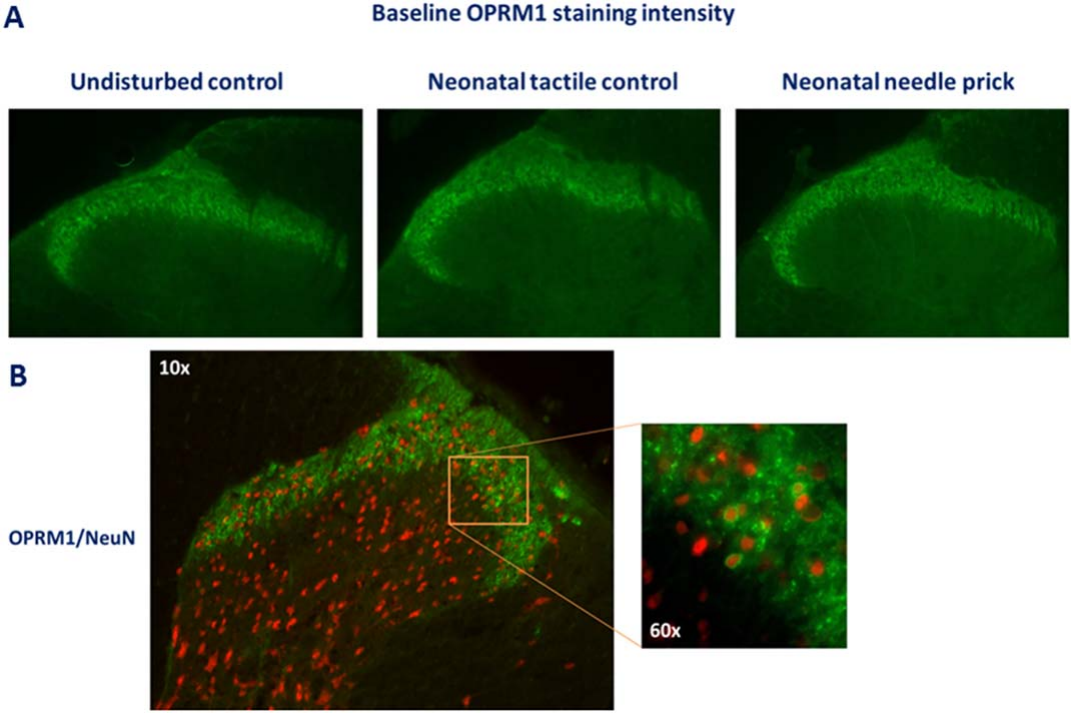
(A) Photographs of OPRM staining in adult animals (unilateral SCDH). Rat pups (littermates) received four noxious needle pricks (needle prick, NP, *n* = 6) or tactile stimulation (tactile control, TC, *n* = 7), or were left undisturbed (UC *n* = 7). No differences in superficial lumbar SCDH OPRM1 staining intensity were observed between groups in adulthood. 3B: Double labelling of OPRM1 (in green) with in red Neuronal marker NeuN, showed exclusive presence of OPRM1 in neuronal membranes. [Color figure can be viewed at http://wileyonlinelibrary.com]

We first tested the effect of neonatal tactile (TC) and needle prick (NP) priming on baseline mechanical sensitivity by assessing paw withdrawal thresholds (PWTs) in adulthood. No significant difference in baseline PWT between TC and NP animals was noted. In addition, baseline mechanical sensitivity did not differ from undisturbed control animals, which were left undisturbed in the early postnatal period (Fig. 2A, effect of procedure: *F*(2, 54) = 1.789, *P* = 0.177). The intensity of OPRM1 staining of the superficial spinal cord dorsal horn (SCDH) was measured as mean gray value, and values of L4 and L5 ipsi‐ and contralateral dorsal horns were pooled as expression did not significantly differ within each group. No significant differences in mean gray value were found between UC, TC, and NP animals (Fig. 2B, effect of procedure: *F*(2, 16) = 0.751, *P* = 0.49). Representative photos of dorsal horn staining of each group are shown in Figure 3A. In order to confirm OPRM1 was only expressed in neurons, we performed double labeling of OPRM1 with neuronal marker NeuN. OPRM1 labeling was exclusively present in the membrane of NeuN‐immunoreactive cells and thus on neurons (Fig. 3B).

### Neonatal Repetitive Tactile and Needle Prick Priming Alters Acute Postoperative Mechanical Hypersensitivity and Decreases Postoperative OPRM1 Staining Intensity in the Superficial Lumbar SCDH 1 and 5 Days After Incision

**Figure 4:**
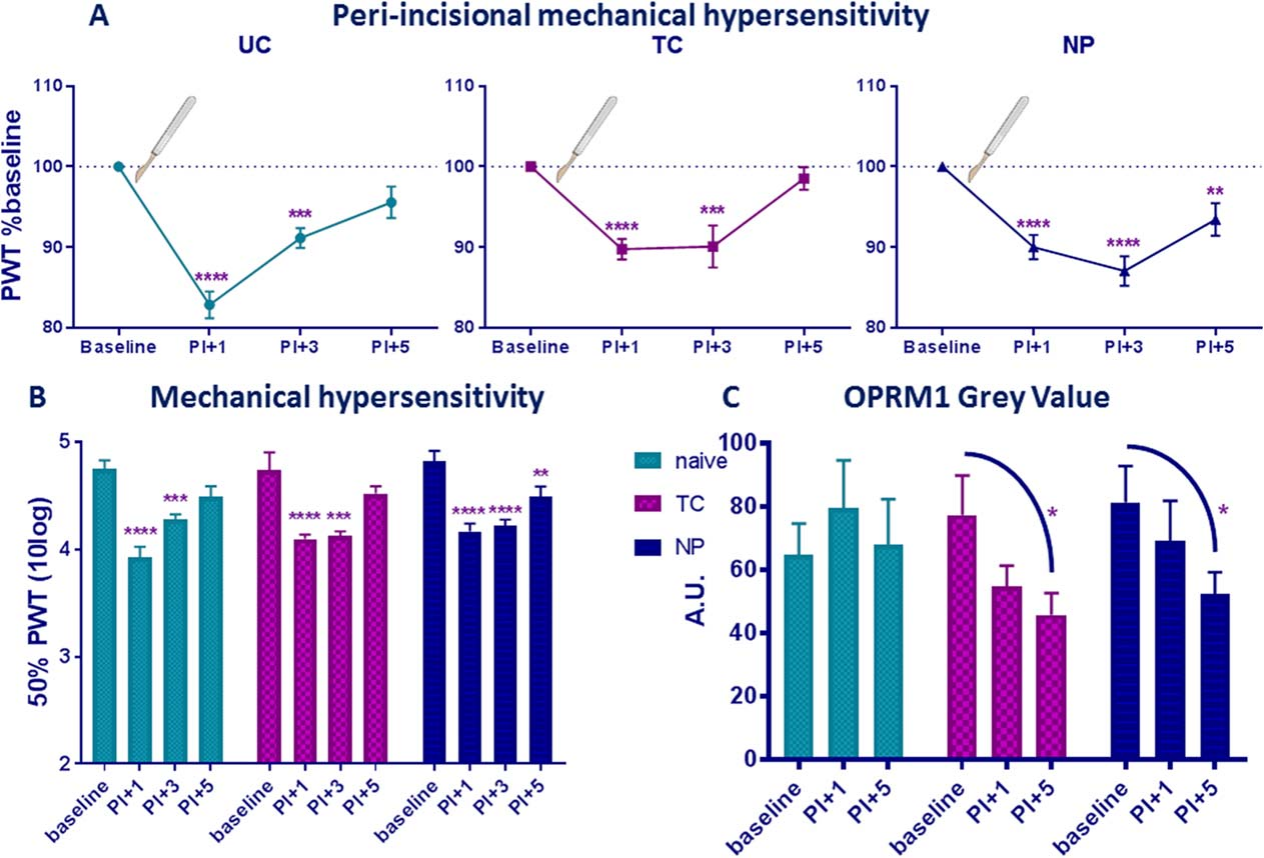
Effect of re‐injury of same dermatome skin on (A) recovery of mechanical hypersensitivity per group expressed as percentage from baseline; (B) mechanical hypersensitivity in all groups; and (C) intensity of superficial (laminae I–III) lumbar SCDH OPRM1 staining expressed as mean gray value in UC, TC, and NP animals. (A) Mechanical sensitivity in adulthood increased in all animals following ipsilateral hind‐paw incision, and 50% PWT was significantly lower 1 and 3 days after incision in all animals (effect of time: *F*(3, 51) = 46.13, *P* < 0.0001). Five days after incision, UC and TC animals were not different from baseline, while NP animals still showed mechanical hypersensitivity (*P* = 0.006). (B) Between group analysis showed no differences between procedures at any time‐point. (C) OPRM1 expression in the superficial SCDH level 4 and 5 did not change over time in UC animals. In TC and NP animals, a significant decrease was observed 5 days post‐incision. Data represented as percentage from baseline (A) or mean ± SEM (B and C); *P < 0.05, **P < 0.01, ***P < 0.001, ****P < 0.0001, within procedure comparisons. [Color figure can be viewed at http://wileyonlinelibrary.com]

Next we tested the effect of re‐injury of the same dermatome 1 and 5 days post‐injury. Behavior was also assessed 3 days post‐injury. All animals showed ipsilateral mechanical hypersensitivity following unilateral hind‐paw incision (Fig. 4A,B, effect of time: *F*(3, 51) = 3,817, *P* = 0.015). Five days after incision, mechanical sensitivity in the UC and TC group had returned to baseline, while NP animals still showed mechanical hypersensitivity compared to baseline (NP baseline vs. PI + 5 *P* = 0.006).

**Figure 5:**
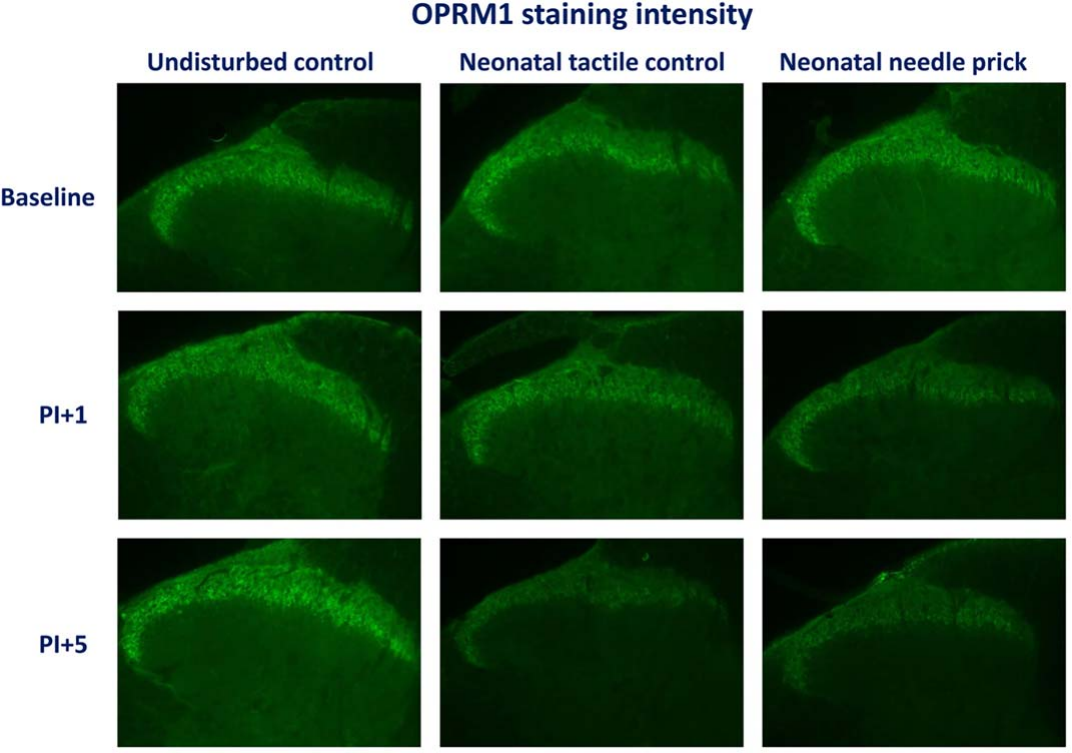
OPRM1 staining intensity in adult animals (unilateral lumbar SCDH). Rat pups (littermates) received four noxious needle pricks or tactile stimulation, or were left undisturbed. In adulthood, animals underwent an ipsilateral hind‐paw incision. Animals were sacrificed and spinal cord L4/L5 tissue was collected and immune‐stained for OPRM1 at 1 and 5 days post‐incision. OPRM1 expression in the superficial lumbar SCDH did not change over time in UC animals. In TC and NP animals, a significant decrease was observed 5 days post‐incision. [Color figure can be viewed at http://wileyonlinelibrary.com]

The intensity of OPRM1 staining (gray value) did not differ from baseline at 1 and 5 days post incision in the UC group (Fig. 4C, effect of time‐point *F*(2, 17) = 0.598, *P* = 0.561). The TC group showed a significant decrease in OPRM1 staining intensity 5 days post‐incision (effect of time‐point *F*(2, 15) = 5.908, *P* = 0.013; baseline vs. PI + 5 *P* = 0.017), as did the NP group 5 days post incision (effect of time‐point *F*(2, 14) = 5.388, *P* = 0.018; baseline vs. PI + 5 *P* = 0.021). Both the NP and TC group did show a trend toward a significant decrease in OPRM1 staining intensity in lumbar SCDH at PI + 1, but this significance did not persist through multiple testing corrections. Figure 5 shows an overview of OPRM1 staining intensity in adult animals.

## DISCUSSION

The semi‐quantitative immune‐cytochemical analysis as in the present study shows that early life repetitive noxious or tactile procedures do not alter baseline levels of OPRM1 staining in the adult lumbar spinal cord dorsal horn. However, our data demonstrates that early life repetitive noxious or tactile procedures lead to a decrease in OPRM1 staining intensity in adult lumbar spinal cord dorsal horn, 5 days after incision as compared to undisturbed controls. This study provides important insights into the anatomical changes to the spinal gate and the role of opioid receptors after neonatal repetitive procedures.

Answering our first research aim, this study shows that early life repetitive noxious or tactile procedures do not alter baseline levels of OPRM1 staining intensity in the adult lumbar superficial spinal cord dorsal horn. With this, our findings add to existing literature on the long‐term effects of neonatal stress and pain and the relation with the developing and adult opioid‐ergic system. Previously, neonatal pain or inflammation has been shown to affect supraspinal areas involved in opioid descending pain modulation (Laprairie and Murphy, 2009; Liu et al., 2004; Yan and Kentner, 2017; Shimada et al., 1990). Neonatal inflammatory pain by carrageenan injection in one hind‐paw on P0 leads to thermal hyposensitivity depending on the central, but not peripheral opioid tone assessed by immune‐cytochemical staining (Laprairie and Murphy, 2009). The same study showed an increased µ‐opioid receptor agonist beta‐endorphin and met/leu‐enkephalin level, and decreased µ‐opioid receptor immunostaining and binding in the PAG (Laprairie and Murphy, 2009). Neonatal intraperitoneal LPS injections at P3 and P5 resulted in an increased mechanical sensitivity in adulthood, associated with a decreased OPRM1 expression in the prefrontal cortex and PAG (Yan and Kentner, 2017). The decreased availability and binding of µ‐opioid receptor in the PAG could lead to decreased morphine sensitivity in adulthood, as shown previously (Laprairie et al., 2008). Likewise, it has been shown that early life capsaicin (P21) leads to acute reduced morphine sensitivity correlated with uncoupling of forebrain µ‐opioid receptors (Liu et al., 2004). In contrast, Shimada and colleagues showed hyposensitivity to thermal stimuli after daily foot‐shocks from P0 to P21, correlated to an increase in morphine sensitivity in adulthood (Shimada et al., 1990). Possibly, a sensitive window exists during which the µ‐opioid receptor coupling is most sensitive to changes. Stimulation at different time points in development leads to opposite effects on morphine sensitivity. Alternatively, the repetitiveness of a painful stimulus during development could alter the opioid system differently.

To answer our second research aim, animals from all three groups (NP, UC, and TC) underwent a (ipsilateral) paw incision. In line with previous studies, we show a prolonged hypersensitivity after adult re‐injury of the ipsilateral paw in neonatal needle prick primed animals (Knaepen et al., 2013; Valverde et al., 2016). Both control groups showed recovery of mechanical sensitivity 5 days post‐incision, while mechanical sensitivity of neonatal needle prick primed animals returned to baseline 7 days after incision. At 1 and 5 days post‐incision, animals were sacrificed and tissue was collected and stained for OPRM1. Previously, it was shown that while OPRM1 mRNA is upregulated in skin after skin incision, OPRM1 mRNA levels remained unchanged in spinal cord (Sun et al., 2015). In line with this, we observed no changes in OPRM1 staining intensity 1 and 5 days post‐incision compared to baseline in neonatally undisturbed animals (UC group) following paw incision. Interestingly, the present study showed that early life repetitive tactile and painful procedures result in a significant decrease in OPRM1 staining intensity post‐incision in adult rats.

In the present study, no differences were observed in OPRM1 staining intensity in adulthood in absence of a second injury to the same dermatome. As most studies did not include behavioral analysis and assessment of mechanical allodynia, but instead used thermal hyperalgesia, it is possible that spinal effects are only unmasked due to the presence of a noxious stimulus like a thermal hot‐plate test or a re‐injury.

A striking observation was the lack of differences in OPRM1 staining between animals undergoing neonatal needle prick versus animals having neonatal tactile priming. Here, neonatal maternal separation might play a role. Indeed, it has been shown that repeated maternal separation (3 h/day between P2 and P14) results into a reduced sensitivity to morphine with a hotplate test in rats (Kalinichev et al., 2001). As maternal separation occurred in our experimental neonatal repetitive needle prick group and our tactile control group, this might blur nociception‐specific consequences.

Abnormal neonatal procedures, be it tactile or noxious, seem to affect OPRM1 expression after a noxious event in adulthood. As it was shown before that OPRM1 expression is correlated to morphine sensitivity (Laprairie and Murphy, 2009; Liu et al., 2004; Shimada et al., 1990), these findings may have clinical implications for optimizing individualized morphine treatment in adult postoperative care. As opioids are still the drug of choice for postoperative pain management in adulthood (Argoff, 2014), early life experience may in part explain differences in sensitivity to opioids. The findings in this study might have impact in understanding the role and potency of morphine analgesia in treatment of chronic postoperative pain. Knowledge of predictors for morphine sensitivity may lead to opting for adapted doses or multi‐modal pain management approaches for post‐operative pain.

Our study is not without limitations. Most importantly, as this is an immunocytochemical study, the measurements are semi‐quantitative. Due to the fixation method of the tissue, we were unable to perform Western blot analysis. Although other techniques might give a better quantification of OPRM1, immunocytochemical analysis has the advantage of providing an anatomical overview. This way, the localization of OPRM1 staining in the spinal cord nociceptive system could be assessed clearly. In addition, using anatomical atlas coordinates allowed us to delineate the superficial laminae (I–III) accurately. With using immunohistochemistry, the specificity and validity of the antibody is important. In this study, we observed intense staining of the cell membrane in the superficial dorsal horn as is the expected location of the OPRM1 protein, corroborating our results, and used negative controls to exclude the possibility of aspecific binding of the antibodies. However, we cannot exclude the antibody stained more than the OPRM1 protein. Another limitation in this study is that we were underpowered to analyze sex differences, but did control for possible effects by using sex as a covariate. Before starting experiments, we performed a literature study, showing no known sex‐specific effects with this specific receptor in the developing CNS (Laprairie and Murphy, 2009; Duhrsen et al., 2013). However, as sex‐specific differences in morphine effectivity in neonatal analgesia have been reported (Laprairie and Murphy, 2009; Laprairie et al., 2008), we cannot completely rule out sex‐specific changes with presence and localization of OPRM‐1.

In conclusion, early life repetitive procedures, whether tactile or noxious, do affect the intensity of OPRM1‐immunoreactivity in the superficial spinal cord dorsal horn after injury in adulthood, without affecting baseline expression. The present translational animal model can be used to test and understand the (changed) potency of morphine after re‐injury in relation to use of neonatal repetitive painful procedures, and in a general context will provide further insights into both spinal and supra‐spinal changes of opioid descending pain modulation.
